# Characterization of Trophoblast and Extraembryonic Endoderm Cell Lineages Derived from Rat Preimplantation Embryos

**DOI:** 10.1371/journal.pone.0009794

**Published:** 2010-03-29

**Authors:** Ilya Chuykin, Irina Lapidus, Elena Popova, Larisa Vilianovich, Valentina Mosienko, Natalia Alenina, Bert Binas, Guixuan Chai, Michael Bader, Alexander Krivokharchenko

**Affiliations:** 1 Max-Delbrϋck Center for Molecular Medicine, Berlin-Buch, Germany; 2 Division of Molecular and Life Sciences, Hanyang University, Ansan, Korea; 3 HD Biosciences Co., Ltd, Zhangjing East Campus, Pudong, Shanghai, China; Emory University, United States of America

## Abstract

**Background:**

Previous attempts to isolate pluripotent cell lines from rat preimplantation embryo in mouse embryonic stem (ES) cell culture conditions (serum and LIF) were unsuccessful, however the resulting cells exhibited the expression of such traditional pluripotency markers as SSEA-1 and alkaline phosphatase. We addressed the question, which kind of cell lineages are produced from rat preimplantation embryo under “classical” mouse ES conditions.

**Results:**

We characterized two cell lines (C5 and B10) which were obtained from rat blastocysts in medium with serum and LIF. In the B10 cell line we found the expression of genes known to be expressed in trophoblast, Cdx-2, cytokeratin-7, and Hand-1. Also, B10 cells invaded the trophectodermal layer upon injection into rat blastocysts. In contrast to mouse Trophoblast Stem (TS) cells proliferation of B10 cells occurred independently of FGF4. Cells of the C5 line expressed traditional markers of extraembryonic-endoderm (XEN) cells, in particular, GATA-4, but also the pluripotency markers SSEA-1 and Oct-4. C5 cell proliferation exhibited dependence on LIF, which is not known to be required by mouse XEN cells.

**Conclusions:**

Our results confirm and extend previous findings about differences between blastocyst-derived cell lines of rat and mice. Our data show, that the B10 cell line represents a population of FGF4-independent rat TS-like cells. C5 cells show features that have recently become known as characteristic of rat XEN cells. Early passages of C5 and B10 cells contained both, TS and XEN cells. We speculate, that mechanisms maintaining self-renewal of cell lineages in rat preimplantation embryo and their in vitro counterparts, including ES, TS and XEN cells are different than in respective mouse lineages.

## Introduction

Embryonic stem (ES) cells are pluripotent cells derived from preimplantation embryos and adapted to *in vitro* cell culture conditions. Currently, the procedure of efficient production of chimaeras, transmission of the genetic changes to the germline and finally the formation of genetically modified animals works efficiently for mouse, but not for other mammalian species. Thus, for better understanding of the pluripotent state in different mammals, it is important to derive ES cell lines from different species and comprehensively describe the requirements for their pluripotency. For this purpose the comparison to mouse Embryonic Stem (mES) cells as a gold standard of pluripotent cells is necessary.

Several types of proliferating cells have been derived from the mouse blastocyst. Mouse ES cells are characterized in detail for the expression of genes maintaining their pluripotent state. Recent studies have suggested that Oct-4, Nanog and Sox-2, in concert with interacting proteins, constitute an autoregulatory, pluripotency network [1], [2]. These marker genes were not found in trophectoderm and extraembryonic endoderm, which represent the first specialized cell types appearing in mouse preimplantation embryos [3]. Trophoblast stem (TS) cells were derived from the mouse blastocyst by cultivation in medium containing FGF4 [4]. Cdx-2 is a transcription factor, which is necessary for the maintenance of these cells [5], [6]. Extraembryonic endoderm (XEN) cells were derived in medium containing serum without additional growth factors. The transcription factors, GATA-4 and GATA-6, were found to be characteristic for this type of differentiation [7], [8]. Thus, three lines derived from mouse preimplantation embryo, have distinct lineage specific markers and requirements for growth factors.

The rat is one of the mostly used laboratory animals for studying the cardiovascular system and many physiological and pathophysiological processes. Multiple laboratory rat lines are available, including outbred, inbred, and genetically modified strains, however, no gene targeting could be obtained due to the difficulties to derive truly pluripotent cell lines [9].

Recently germ-line competent rat ES cells were successfully derived by application of inhibitors of signaling pathways mediated by kinases ERK and GSK3β [10], [11]. These studies uncovered a powerful tool for the derivation of ES cells from different mammalian species. Interestingly, previous attempts to isolate pluripotent cell lines by conventional cultivation of rat blastocysts in the medium suitable for mouse ES cells were unsuccessful. Also, the lineage identity of cells obtained from the rat preimplantation embryo without inhibitors remained questionable. It was shown that these so-called rat ES-like cells are LIF-dependent, and positive for alkaline phosphatase and SSEA-1 [12]–[15]. Oct-4 expression in rat ES-like cells was reported by one research group [12], but not by others [15]. It was proposed, that derivation of ES cells from rat blastocysts is limited by the decrease of Oct-4 expression during the process of outgrowth [14]. In our laboratory, several cell lines from different strains of rats have been obtained. These rat ES-like cell lines were shown to be dependent on LIF, and positive for classical pluripotency markers SSEA-1 and alkaline phosphatase [15]. Nevertheless, it was not possible to get chimaeras from these cells after blastocyst injection.

In this study we asked the question, why cell lines obtained from rat preimplantation embryos, namely C5 and B10, did not contribute to chimaeras. We characterized C5 and B10 cells by analyzing the expression of marker genes for embryonic stem cells and extraembryonic lineages. We showed that B10 and C5 cell lines consist of cells committed to 2 independent lineages: trophectoderm (as revealed by expression of Cdx-2, cytokeratin-7, and Hand-1) and endoderm (positive for GATA-4, -6, laminin B, and collagen-4). Our data also show, that Cdx-2 and GATA-4 expressing lineages are formed very early during the process of outgrowth from rat blastocysts. Interestingly, the derivation and propagation of rat TS cells did not require the cell culture conditions used for mouse Trophoblast Stem (mTS) cells and occurred in the standard cell culture medium. Rat endodermal cells also revealed peculiar features, such as dependence on LIF and the expression of pluripotency markers, SSEA-1 and Oct-4. Presumably, the inhibitors of MEK1 and GSK3β kinases which have been successfully used to derive the pluripotent rat ES cells [10], [11] are necessary to suppress TS and XEN lineage formation which can grow under classical ES-cell conditions (medium with serum and LIF) in this species.

## Results

### Cell morphology and pluripotency marker gene expression in cell lines derived from rat blastocysts

**Figure 1:**
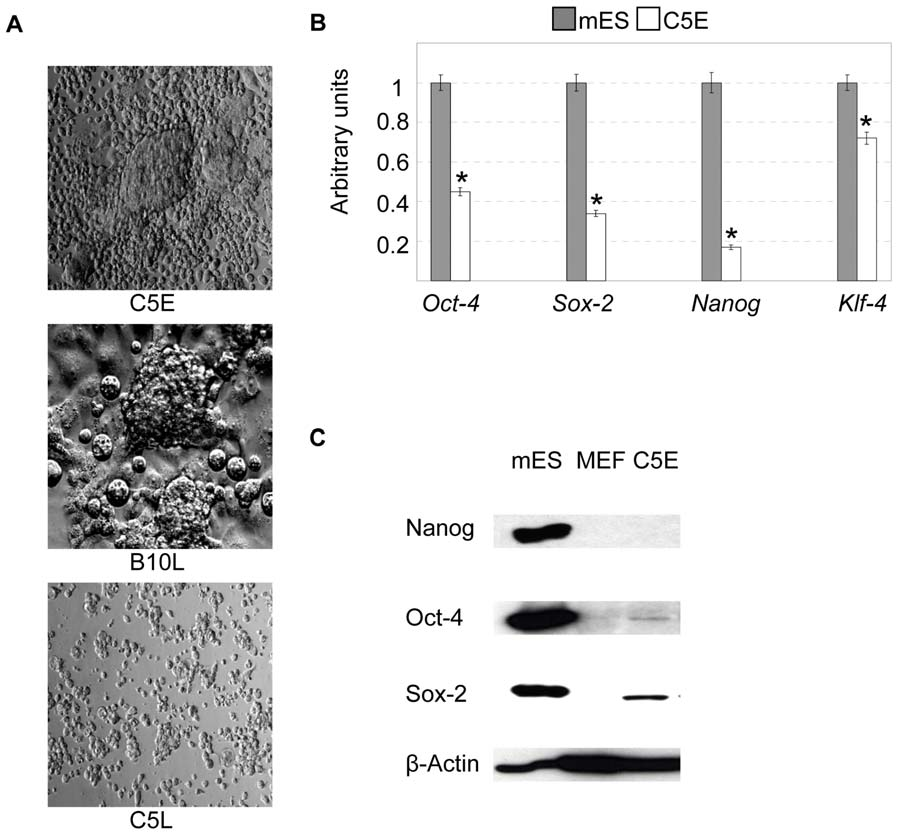
Morphological features and expression of pluripotency genes in cell lines derived from rat blastocysts. (A): Morphology of cell line C5E, B10L and C5L, photos were made with 10× objective (C5E and C5L) and 20× objective (B10L). (B): Real-time PCR analysis of *Oct-4*, *Sox-2*, *Nanog*, *KLF-4* gene expression in C5E and mES cells. The data were normalized by the amount of β-actin and plotted against the expression level in mES cells set as 1.* p<0.01 *vs.* mES cells; AU, arbitrary units. (C) Western-blot analysis of Oct-4, Nanog and Sox-2 protein levels in mES cells (positive control), MEF (negative control) and C5E.

The experiments were performed with the previously characterized B10 [15] and C5 cell lines, obtained from the inbred Wistar Kyoto (WKY) and the outbred Sprague Dawley (SD) strain of rats, respectively. These cell lines were employed at early and late passages. In this work we characterised *C5 early* (passages 5–7) (C5E), *C5 late* (passages 20–30) (C5L), and *B10 late* (passages 30–60) (B10L) cell lines. C5L was obtained after expansion of a single cell, whereas B10L was derived by the expansion of individual clumps of tightly growing cells. B10L consisted of tight clumps and large flattened cells, whereas C5L consisted of rounded cells which were loosely attached and did not form tight clumps (Figure 1A). In C5E one can see morphological features, also present in both C5L and B10L: cells residing in round colonies (clumps), large flattened cells, and small round loosely attached cells with endodermal morphology, which do not form clumps (Figure 1A). We first evaluated the expression of pluripotency genes in C5E. Real time PCR revealed lower levels of *Oct-4*, *Sox-2*, *Nanog* and *Klf-4* in C5E line in comparison to mES cells, which were taken as a positive control. In accordance with real-time PCR data only very low levels of Oct-4 and Sox-2 were found in C5E cells by western blot, whereas Nanog was not detectable by this method (Figure 1C). We were interested to look in details, which cell types are present in these cell lines, therefore we described in detail B10L and C5L as these lines were cloned from single clump (B10) or cell (C5) and contained less diverse number of cell types.

### B10L reveal signs of trophectoderm differentiation

**Figure 2:**
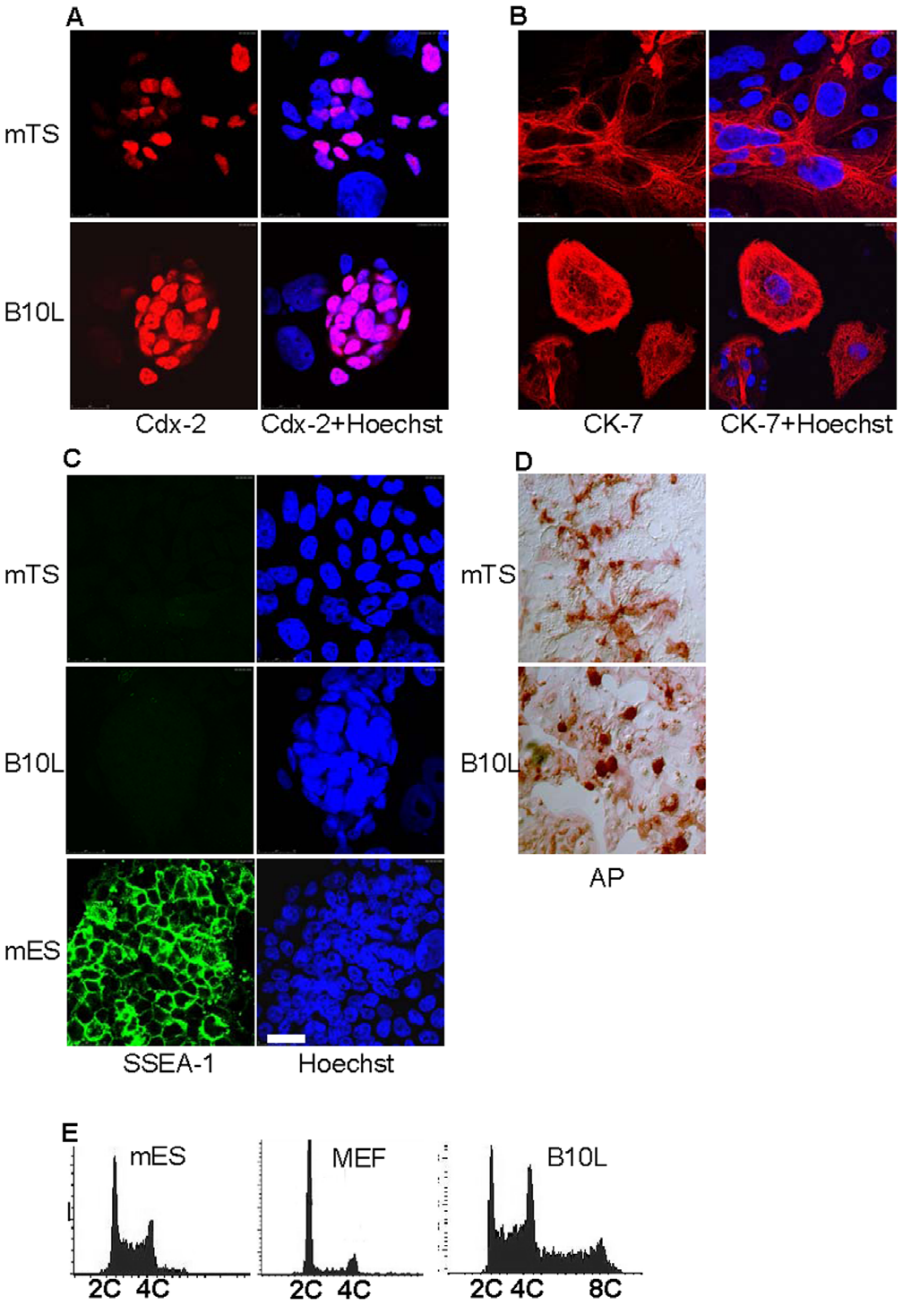
Markers of trophectodermal lineage in B10 cell line. Immunostaining and confocal microscopy for Cdx-2 (A), Cytokeratin-7 (CK-7) (B), and SSEA-1 (C) in mTS, B10, and mES cells, scale 25 µm. Nuclei were stained with Hoechst 33342. (D): Alkaline Phosphatase (AP)-test in mTS and B10L cells. (E): Cell cycle phase distribution in mES cells, MEF and B10 cells. 2c, diploid, 4c, tetraploid, 8c, octaploid cells.

Since large flattened cells in the B10L cell culture (Figure 1A) were similar in morphology to giant trophoblast cells, we analysed the expression of the trophectoderm marker Cdx-2 in B10L. This factor induces trophectodermal differentiation in mES cells and is necessary to support self-renewal of mouse Trophoblast Stem (mTS) cells [5], [6]. Therefore, a previously characterized line of mouse mTS cells [4] was taken as positive control. Immunofluorescence analysis revealed Cdx-2 expression in both mTS and B10L cells (Figure 2A). Noteworthy, the characteristic pattern of 

Cdx-2 expression was observed in B10L. Cdx-2 staining was predominantly observed in small cells residing in clumps, but was not detected in large nuclei of differentiated cells (Figure 2A). Giant cells in the populations of B10L and mTS cells expressed the epithelial marker cytokeratin-7 (Figure 2B), which was also found in trophectoderm [5]. Previously, rat ES-like cells of low passage number were described as positive for alkaline phosphatase (AP), Oct-4 and SSEA-1 [12], . However, neither B10L nor mTS cells expressed SSEA-1 (Figure 2C), whereas mES cells, which were taken as positive control, were SSEA-1 positive in the same conditions of immunostaining (Figure 2C). Oct-4 expression was not observed in B10L cells (Figure S1A). Groups of cells positive for AP-activity were detected in both, B10L and mTS cell lines (Figure 2D). All clumps containing small cells in B10L were strongly AP-positive (Figure 2D). In order to confirm the presence of polyploid cells typical for differentiated trophoblast in B10L we performed a cytometric analysis of cell cycle phase distribution. As it is expected for fastly dividing cells, the majority of mES cells were in the S-phase of the cell cycle in contrast to MEF (mouse embryonic fibroblasts), which were mostly in G1 phase (Figure 2E). The profile of cell cycle phase distribution of B10L was similar to mES cells with high proportion of S-phase cells, but unlike mES cells, B10L contained a significant amount of polyploid cells (Figure 2E). Presumably, the small cells residing in clumps represent a Cdx-2-positive population of rat TS cells which constantly produce the differentiated progeny of giant polyploid trophoblast cells. E-cadherin is necessary for the formation of the trophectodermal layer in the preimplantation embryo. However, during differentiation of the trophoblast and the acquisition of an invasive phenotype trophoblast cells loose the expression of E-cadherin [16]. The pattern of E-cadherin staining in B10L cells was similar to Cdx-2: in compact clumps cells expressed E-cadherin, whereas in giant differentiated cells the expression of E-cadherin was lost (Figure S1B). Sox-2 was expressed in the extraembryonic ectoderm and mouse TS cells [17]. We checked the expression of Sox-2 at the protein level and found no expression in B10L cells (Figure S1C).

### B10 cells injected into rat blastocyst invade the trophectodermal layer

**Figure 3:**
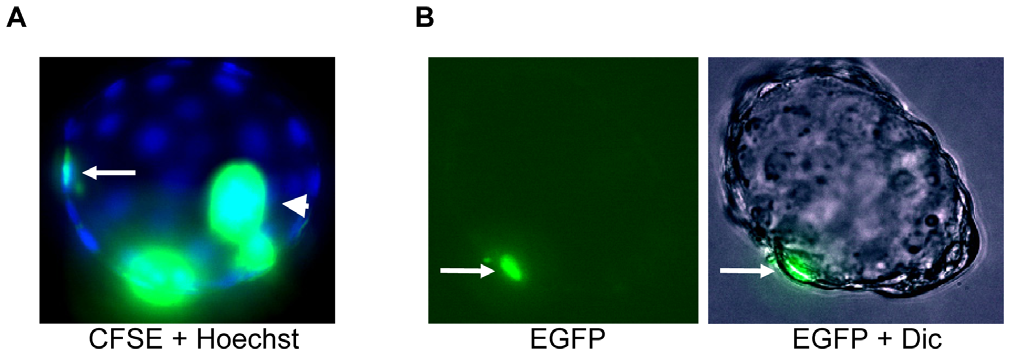
B10 cells contribute to the trophectodermal layer upon injection into rat blastocyst. (A): Rat blastocyst containing CFSE- labelled cells in the trophectodermal layer (arrow), merged image of carboxyfluorescein succinimidyl ester (CFSE) fluorescence and Hoechst 33342 is shown, arrowhead indicates non-included cells in blastocoel. (B): Rat blastocyst with B10 cell expressing EGFP in the trophectoderm layer (arrow), fluorescent image and merged image of EGFP with differential interference contrast (Dic) is shown. Photos were taken with 40× objective.

To further confirm the commitment of B10L to the trophectodermal lineage we fluorescently labeled the cells and injected them into the cavity of early SD rat blastocysts. In the first approach labeling with the fluorescent dye CFSE was performed. In the second experiment, we used B10L cells stably expressing EGFP-NLS upon infection with a lentivirus. In both experiments we observed the inclusion of labeled B10L cells into the trophectoderm layer (Figure 3A, B; Figure S2). The integration efficiency is shown in Table 1.

**Table 1:** Incorporation of labelled (CFSE and lentivirus bearing EGFP) B10L cells rat blastocysts.

Method of cell introduction	No. of embryos used	Time of in vitro culture (hr)	No. of incorporated cells into trophectoderm per injected embryos
Injection of 5–7 B10L cells into blastocyst	37	24	11/37 (29.7)%

### The proliferation of B10L is independent of FGF4

**Figure 4:**
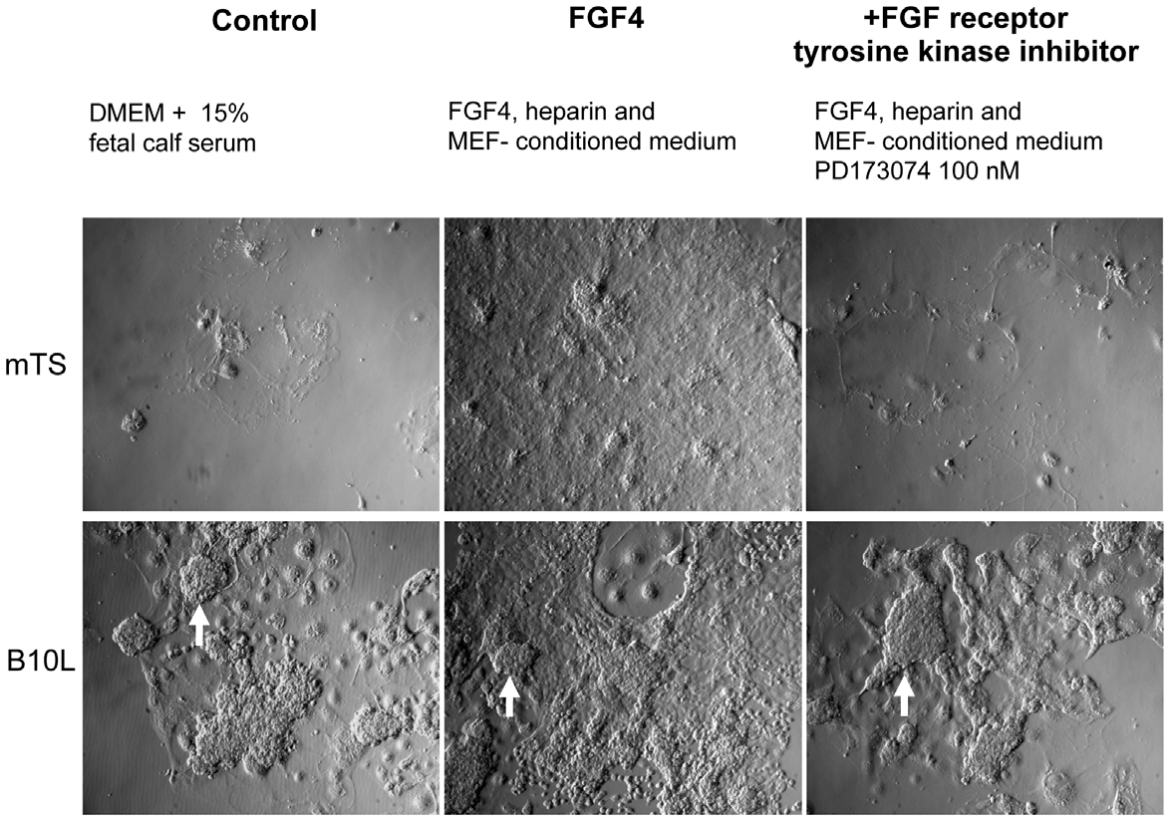
The proliferation of B10L cell line is FGF4-independent. (A): Morphology of B10 cells and mTS cells under different culture conditions, photos were made with 10× objective. Characteristic B10L morphology is shown by a white arrow.

Derivation and propagation of diploid mouse TS cells strictly relies on the stimulation with FGF4 and conditioned medium from MEF, as these conditions allow the proliferation and inhibit the differentiation of Cdx-2 - positive mouse TS cells to other types of trophoblast cells [4]–[6]. In order to clarify if the growth of rat B10L cells is also dependent on FGF4 we cultivated B10L and mTS cells in the following conditions: (1) in basal medium used to propagate B10L (2) in the presence of FGF4, heparin and conditioned medium from MEF, (3) as in (2) but with addition of the FGF receptor 1 (FGFR) inhibitor, PD173074. Cells were seeded at low density (5×10^3^ cells/cm^2^), cultivated for 5 days and then replated in equal density and cultivated for 5 days more. The propagation of mTS cells was achieved in conditions (2) only. In the absence of FGF4 or in the presence of PD173074 all mTS cells were differentiated (Figure 4). In contrast to mTS cells, B10L grew in all 3 conditions (Figure 4), maintaining Cdx-2 expression (Figure S3). Therefore, we conclude that proliferation of B10L cells is independent of FGF4.

### Expression of extraembryonic endoderm markers in the rat C5L cell line

**Figure 5:**
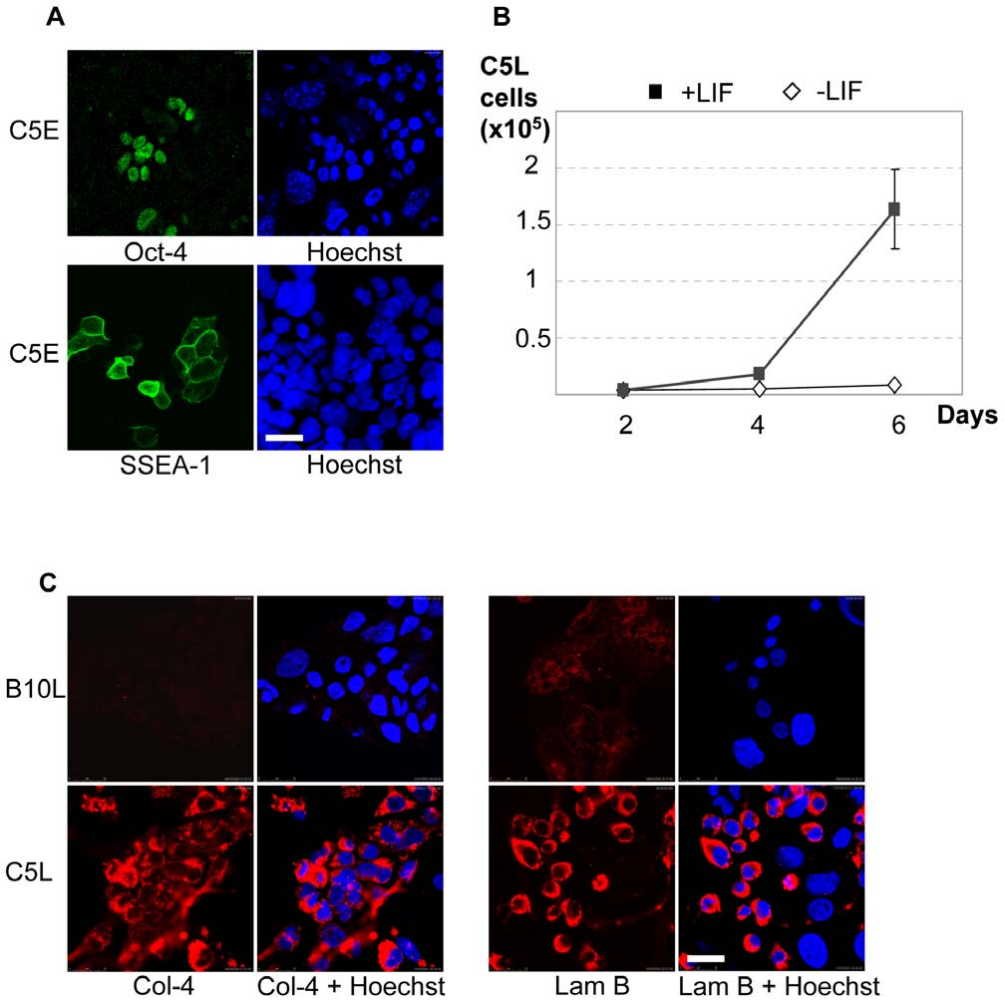
Expression of endoderm markers in C5 cells. (A): Immunostaining for SSEA-1 and Oct-4 in C5E cells, scale 25 µM. (B): Growth curves of C5L cells cultivated with or without LIF. (C): Immunostaining for laminin B (Lam B) and collagen-4 (Col-4) in C5L and B10L cell lines, scale 25 µm.

Recently, we derived from rat blastocysts cells that co-express Oct-4, SSEA-1 and the endoderm transcription factor GATA-6 and require LIF for colony formation [18]. Since the C5 cells of early passage (Figure 1A) and the B10 cells of early passage (not shown) also show the characteristic endodermal morphology, we tested whether the C5 cells exhibit similar properties. By contrast to B10L cells, tight clumps and large flattened cells were absent in C5L cells (Figure 1A). Depending on the presense or absence of MEF feeders, the morphology of C5L varied from epithelial to loosely attached rounded cells (Figure S4A). In accordance with our previous data [18] and real-time PCR (Figure 1B), we revealed in C5E line cells which were positive for pluripotency markers Oct-4 and SSEA-1 (Figure 5A). To verify the dependence on LIF, we used the population of C5L, that was devoid of clumps and differentiated trophoblast, characteristic for B10L. As expected [18] we found, that proliferation of C5L cells was drastically dependent on LIF (Figure 5B, Figure S4). Stimulation of C5L cells with LIF resulted in accumulation of STAT-3, phosphorylated on tyrosine 705. (Figure S4B) This result is in sharp contrast to B10 cells, which can be cultivated without LIF (data not shown). Finally, we analyzed the expression of extraembryonic endoderm markers in C5L. In agreement with the previous analysis of rat endoderm cells [18], C5L cells expressed collagen-4 and laminin B (Figure 5C). Both proteins are components of the basal membrane produced by parietal endoderm [19]. By contrast B10L cells were only weakly stained for laminin B and did not express collagen-4. Thus, the expression of these markers was characteristic for C5L, but not for the trophectoderm-committed line B10L (Figure 5C).

### Cell lines derived from the rat blastocyst contain lineages of cells committed to endoderm and trophectoderm

**Figure 6:**
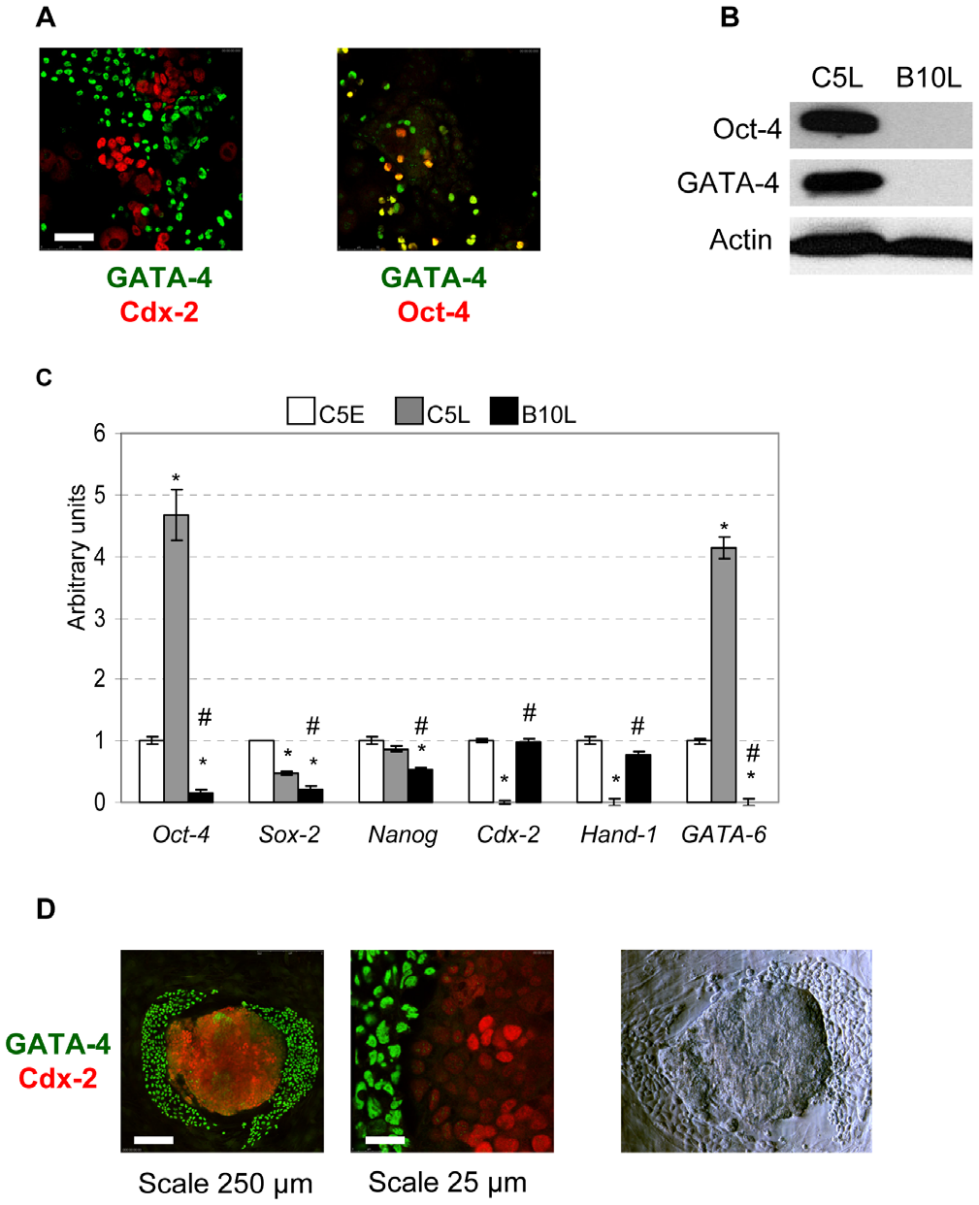
Endoderm and trophectoderm-committed cells, derived from rat blastocysts. (A): Expression of Cdx-2 and GATA-4 (left panel) and GATA-4 and Oct-4 (right panel) in C5E early cells, scale 50 µm. (B): Western blot analysis of Oct-4 and GATA-4 expression in B10L and C5L cells. (C): Real-time PCR analysis of *Oct-4*, *Sox-2*, *Nanog*, *Cdx-2*, *Hand-1*, *GATA-6* gene expression in C5E, C5L and B10L cells. These data were normalized by the amount of *β-actin* and plotted against the expression level in C5E cells set as 1, * p<0.01 vs. C5E, # p<0.05 B10L vs. C5L; AU, arbitrary units. (D): immuno-staining for Cdx-2 and GATA-4 proteins in 6 day outgrowths of rat blastocysts, right panel: photo of the same rat outgrowth made on light microscope with Hoffmann modulation contrast, 20× objective.

C5E line contains cells of two morphological types, which were seen separately in C5L and B10L cell lines (Figure 1A). Therefore we analysed the expression of early endodermal (GATA-4) and trophectodermal (Cdx-2) markers in the C5E cell line. Cells expressing Cdx-2 and GATA-4 were detected in C5E, however no cells coexpressing both transcription factors could be found (Figure 6A). In contrast, Oct-4 and GATA-4 coimmunostaining resulted in partially overlapping patterns (Figure 6A). By western-blotting we confirmed, that C5L but not B10L expressed GATA-4 and Oct-4 (Figure 6B). We further compared by real-time PCR the expression of genes, shown to be necessary to maintain pluripotency, (*Oct-4*, *Sox-2*, *Nanog*), trophectoderm (*Hand-1*, *Cdx-2*), and the marker of endoderm (*GATA-6*) in C5E, C5L, and B10L. Both trophectoderm-specific genes were expressed in C5E and B10L, but were completely absent in C5L (Figure 6C), confirming the absence of trophectoderm-committed cells in C5L. On the other hand, high levels of *GATA-6* together with *Oct-4* were detected in C5L (Figure 6C). In accordance to our western blot data (Figure S1C) very low amount of Sox-2 mRNA was detected in B10L cells (Figure 6C) suggesting that this gene was absent in the B10L line. Except Oct-4, the tested pluripotency genes were expressed slightly lower in C5L and B10L in comparison to C5E, but none of the rat lines reached the level of mES cells for these genes (Figure 6C, Figure 1B, C). By immunofluorescence analysis we found no expression of Nanog in B10L and C5E cells, whereas clear nuclear staining was seen in mouse ES cells and rat blastocyst (Figure S5). The fact that early but not late C5 cells exhibited a mixed morphology and gene expression pattern prompted us to investigate whether the segregation into the endoderm and trophectoderm cell types occurs upon the plating of rat blastocyst on feeder cells in mouse ES medium. Therefore, we placed rat blastocysts on MEF cells. After 6 days, outgrowths were formed. Morphologically, one could distinguish 2 types of cells: cells in smoothened clump of ES-like morphology in the center and epithelial-like cells on the edge of the outgrowth (Figure 6D). We performed immunostaining for Cdx-2 and GATA-4 and found cells in smoothened clumps to be Cdx-2-positive whereas GATA-4-positive cells were in the periphery. Thus, similar to early passage of C5 cells, we observed the non-overlapping pattern of Cdx-2 and GATA-4 expression in outgrowths derived from rat blastocysts. In medium containing bovine serum and LIF we derived 9 cell lines from rat preimplantation embryos at morula and blastocyst stages from Sprague Dawley, Fisher and hybrid Fisher x Wistar Kyoto rats (Table 2). The initial formation of clumps, expansion of cell lines showing the features of trophoblast and extraembryonic endoderm is shown in Figure S6. From the C5E cell line we have additionally derived 4 stable TS-like subclonal cell lines showing the same morphology as the B10L line (Figure S7 A) and expression of Cdx-2 (Figure S7 B, C). This demonstrated the potential to derive stable lines of both extraembryonic lineages from a single rat preimplantation embryo under the same culture conditions.

**Table 2:** Overall efficiency of derivation of cell lines from rat preimplantation embryos.

Strain of donor embryo	Attachment rate (%)		Efficiency of clump formation		Number of derived cell lines
	morula	blastocyst	morula	blastocyst	
Sprague Dawley	6/8 (75%)	24/33 (73%)	4/6 (67%)	16/24 (67%)	2 early blastocyst 1 morula
Fisher		5/5 (100%)		5/5 (100%)	4 blastocysts
Fisher ♂ Wistar Kyoto ♀	7/20 (35%)		5/7 (71%)		2 morula

## Discussion

We provide evidence, that the rat blastocyst derived B10L cell line represents a population of TS-like cells. This conclusion is supported by two observations: first, labelled B10 cells invaded the trophectoderm layer upon injection into blastocysts (Figure 3). Second, the transcription factors Cdx-2 and Hand-1, which are established trophoblast markers in mice [5], [6], are expressed in B10L cells confirming their trophectodermal identity (Figure 2A, Figure 6C). Our results coincide with previous findings. The expression of Cdx-2 was shown by *in situ* hybridization in cells derived from rat blastocysts [14]. Also the contribution of cells obtained from rat embryos to extraembryonic lineages upon blastocyst injection has recently been reported [13]. Similar to mTS cells, B10L do not express the pluripotency markers, Oct-4, Nanog, and SSEA-1 (Figure 1B, Figure 2C, Figure 6C, Figure S5). Although B10 cells expressed AP, a marker that can identify embryonic stem cells [12] AP is clearly a marker of limited specificity since we found it also in mTS cells. Hence, AP-activity is not a distinguishing feature of rat ES cells. Importantly, the propagation of B10 cells occurred without FGF4, and even in the presence of the FGFR inhibitor, PD173074. At the same time an mTS cell line was able to proliferate only in the presence of FGF4 (Figure 4). These data allow us to conclude, that proliferation of rat TS-like cells is uncoupled from FGF4. This peculiar feature distinguishes rat TS-like cells from mTS cells (since FGF4 is necessary for the derivation of mTS cells from the embryo and for their proliferation in culture [4]).

Recently, extraembryonic endodermal cell lines from rat blastocysts were obtained and comprehensively characterized by marker gene expression [18]. In addition to GATA-6, these cells were shown to express Oct-4 and SSEA-1, which are known to be typical markers of mouse ES cells. Here we confirm these data and show, that C5L consists of cells committed to extraembryonic endoderm (Figure 5A). We show, that these cells express GATA-4 (at the protein level) and GATA-6 (at the level of mRNA) (Figure 6A, B, C). These factors are involved in the specification of the endodermal lineage in mice [20]. Moreover, C5L, in contrast to B10L, express the extracellular matrix proteins, laminin B and collagen-4 (Figure 5A), which are components of Reichert's membrane produced by parietal endoderm [19]. A second interesting property of the C5 cells is their dependence on LIF (Figure 5C), which is similar to the recently characterized endodermal cell lines from rat blastocysts [18]. This feature was not previously shown for mouse XEN cells since their derivation occurred without addition of LIF [7]; [20]. Most interestingly and similar to the described rat extraembryonic endoderm cells [18], [21] the C5 cells highly expressed Oct-4 at the protein and mRNA level (Figure 6 B, C). At early passage, when the C5 cells consisted of a mixture of cells with either trophectodermal or endodermal markers, overlapping patterns of expression were seen for Oct-4 and GATA-4, but not for GATA-4 and Cdx-2 (Figure 6A), verifying the specific expression of Oct-4 in rat endoderm lineage. Although Oct-4 was previously suggested to be a limiting factor during the procedure of isolation of rat ES cells [14], it appears that in addition to its function as pluripotency factor, it might have a role for the self-renewal of rat XEN cells. It has been shown that increase in Oct-4 expression induces endodermal and mesodermal differentiation in mES cells [22]. However in endoderm cells obtained from mouse embryos [7] or from mES cells by overexpression of GATA-4 and GATA-6 [8], the expression of Oct-4 and SSEA-1 was not shown. Furthermore, it was shown that endodermal differentiation of mES cells caused by overexpression of GATA-4 and GATA-6 correlates with downregulation of Oct-4 [20]. Therefore, it appears, that the expression of SSEA-1 and Oct-4 (both are markers of pluripotent mES cells) is a characteristic of rat XEN cells. Therefore, it is interesting to mention the difference in *in vitro* differentiation potential of epiblast tissue from mouse and rat embryos. Rat, in contrast to mouse epiblast produce parietal endoderm cells [23]. The genes constituting a pluripotency network with Oct-4 in mES cells, Sox-2 and Nanog [2], are not highly expressed in C5L (Figure 6C, Figure S5), therefore rat XEN cells represent an interesting model to study, which genes are regulated by Oct-4 in this lineage.

Little or no Nanog expression was detected on mRNA and protein level in either C5L or B10L cell lines, although it was readily detected in rat blastocysts by immunostaining (Figure 1B, C; Figure 6C; Suppl Fig. S5). Obviously, the pluripotent Nanog-expressing population of cells is not maintained during the procedure of rat cell lines derivation in the conditions used for mouse ES cell derivation. We suggest, that a reporter gene construct, driven by the Nanog promoter in transgenic rats, similar to what was published for mouse [24], would help to enhance the selection process of germline-competent ES cells during the derivation from rat preimplantation embryos and induced pluripotent rat stem cells.

Another interesting aspect concerns the block of the trophectoderm differentiation in ES cells. Published data support the notion, that mES cells do not normally undergo trophectoderm differentiation without genetic manipulation, e.g. ectopic expression Cdx-2 or repression of Oct-4 [6], [25]. This might be connected with DNA methylation-mediated silencing of a trophoblast-specific transcription factor Elf-5 blocking the trophectoderm differentiation in mES cells [26]. Whether this restricting mechanism exists in the rat preimplantation embryo and rat ES cells is unknown. One possible approach to clarify this is to analyse whether rat ES cells obtained in the presence of inhibitors of MEK and GSK3β kinases [10], [11] are able to undergo trophoblast differentiation under TS culture conditions or whether this type of differentiation in rat ES cells is suppressed similar to mouse ES cells. In this work we show, that during attempts to isolate rat ES cells in the cell culture medium suitable for the ES cell derivation in certain strains of mice (such as 129 mice), proliferating pools of TS and XEN populations of cells are specified. Importantly, during the process of outgrowth from rat blastocysts TS cells are produced without FGF4, which is not the case for mouse TS cells [4]. As specification of the trophectoderm lineage is the first differentiation appearing *in vivo* in mammalian embryo [3], it is very likely, that the process of rat ES cells derivation with the signaling pathway inhibitors [10], [11] involves the block of trophectoderm differentiation. Previously the participation of kinase ERK2 was reported to be important for the formation of trophoblast tissues, extraembryonic ectoderm and the ectoplacental cone during mouse development [27]. The efficient application of MEK and GSK3β kinase inhibitors for the derivation of pluripotent cells uncovers the fundamental mechanisms concerning the role of the respective pathways in the differentiation commitment to the pluripotent lineage in rodents. In this study we report that rat TS and XEN lineages are different from the respective mouse lineages in the requirements for growth factors and expression of marker genes (Oct-4 and SSEA-1 in rat XEN cells). We speculate, that mechanisms maintaining self-renewal of cell lineages in the preimplantation rat embryo and their in vitro counterparts, including ES, TS and XEN cells are different from mouse and require further detailed description.

While this manuscript was under review, Debeb et al. [18] and Galat et al. [28] published a characterization of rat extraembryonic endoderm precursor cell lines, that revealed the dependence on LIF and the expression of Oct-4. The authors further show data on the developmental potential of those cell lines postimplantation. Galat and coauthors demonstrate, that rat extraembryonic endoderm precursor cells colonize yolk sac and contribute to trophoblast lineages of post-implantation embryos following transfer to surrogate mothers. In the present study we reveal that the early passages of cells derived from rat preimplantation embryos contain two distinct cell lineages showing features of trophoblast stem-like cells and extraembryonic endoderm-like stem cells. A detailed comparison of the cell lines derived in our group with the published ones [18], [28] will be an important subject of future studies.

## Methods

### Ethics Statement

All experiments were performed according to the guidelines for the human use of laboratory animals by the Max-Delbruck Center and were approved by local German authorities with standards corresponding to those prescribed by the American Physiological Society.

### Cell culture

B10 and C5 cell lines were derived from the inbred WKY and outbred SD rat strains respectively. B10 cell line was described [15]. Experiments were done on *C5 early* (passages 5–7) (C5E), *C5 late* (passages 20–30) (C5L), and *B10 late* (passages 30–60) (B10L) cell lines. C5L was obtained after singe cell cloning. B10L was obtained by amplification of a single tight clump. The B10 cell line was cultivated without feeders. C5 cells were cultivated with or without feeders, as indicated. As feeder cells, mitomycin C-treated mouse embryonic fibroblasts (MEF), passages 3–5, isolated from 14-day old mice (NMRI strain) embryos were used. Mouse feeder-free ES cell line E14tg2a (passages 30–60) was used as positive control in several experiments. B10 cells were grown in the medium, consisting of DMEM (Sigma) with 15% fetal calf serum (Invitrogen) supplemented with nonessential amino acids, glutamate, sodium pyruvate, β-mercaptoethanol, penicillin-streptomycin. C5 cells were grown in the same medium supplemented with ESGRO (Chemicon), 1000 U/ml. C5 and B10 cell lines were replated every 2–5 day after 5 minutes of trypsinization. The mouse trophoblast stem cell line, GFP-EXE, was a kind gift from the lab of Janet Rossant and was cultivated according to the conditions published on the web site: http://www.sickkids.ca/rossant/protocols/TScells.asp. For alkaline phosphatase staining cells were fixed in 4% paraformaldehyde, washed with PBS, and then incubated for 30 minutes in Tris-Maleate buffer containing FAST-RED TR, F2768 (Sigma) and Naphtol-AX-MX, N5000 (Sigma).

### Animals

Female Sprague-Dawley (SD) rats (60-day-old) were obtained from a commercial animal breeder (Charles River/Sulzfeld). The rats were kept at a temperature of 21±2°C in a 12 hr light/dark cycle (lights on 6.00 a.m.–6.00 p.m.) with a humidity of 65±5%.

### Isolation of blastocysts

For production of embryos at blastocyst stage females SD rats were mated with males of the same strain and on the following morning (day 1 of pregnancy) examined for a plug. Pluged females were sacrificed at 12 a.m. on day 5 of pregnancy and blastocyst stage embryos were recovered by flushing the excised uterine horns with M2 medium (Sigma) [29].

### Microinjection of cells into blastocysts

B10 cells were labelled with fluorescent substances (5 µM CFSE) or by infection with lentivirus, containing EGFP (kind gift of S. Diecke and D. Besser, MDC). For the enrichment of the EGFP-positive population B10L-EGFP cells were additionally FACS-sorted on BD FACSAria sorter. Blastocyst injection was carried out by injecting 5–7 B10L cells into cavity of host blastocysts of SD rats. After injection, blastocysts were cultured *in vitro* overnight in M16 medium in 4-well culture dishes (Nunc) under 5% CO_2_ in air at 37°C [30], and then analysed under the fluorescence microscope DMI6000B (Leica).

### Rat blastocyst outgrowths

Blastocysts of SD rats of day 4.0–4.5 of development were plated in 4-well dishes (Nunc) on coverslips covered with MEFs in ES medium containing ESGRO (1000 U/ml). Embryos were undisturbed until attachment (2–3 days) and afterwards the medium was changed daily. After 6 days the outgrowths were fixed with 4% paraformaldehyde and subjected to immunostaining.

### Flow cytometry analysis of cell cycle phase distribution

For cytometric analysis of DNA content, 5×10^5^–10^6^ cells were harvested, washed with PBS, resuspended subsequently in 100 µl of solution A (0.25 M sucrose, 40 mM sodium citrate pH 7.6] and 400 µl of solution B (in PBS: 0.5% NP-40, 0.5 mM EDTA, 100 µg/ml RNaseA [Sigma], 40 µg/ml propidium iodide [Sigma]), and incubated for 15 min at 37°C. Probes were analyzed by flow cytometry on a BD Calibur.

### Immunocytochemistry

Cells were cultured on MEF or gelatine-coated coverslips, fixed in 4% paraformaldehyde (Sigma), permeabilised in 0.5% Triton-X-100 (Sigma), incubated in blocking solution (5% BSA in PBS) for 1 hour. Blocked samples were incubated for 1 hour at room temperature or overnight at 4°C with primary antibodies diluted in blocking solution 1∶50 (anti-Oct-4, sc-5279, anti-GATA-4, sc-9053 [Santa Cruz Biotechnology Inc], anti-SSEA-1, MC-480, anti-laminin B2, D18, anti-collagen type 4, M3F7, anti-CD9, B2C11, [Developmental Studies Iowa Hybridoma Bank], anti-cytokeratin-7, MAB3236 [Millipore], anti-Cdx-2, Cdx-2-88 [BioGenex], anti-Nanog, ab21603, [Abcam], anti-E-cadherin, 610182, [BD Bioscience]. Then samples were incubated for 1 hour with secondary antibodies (goat anti-mouse Alexa 647, A21237, goat anti-mouse IgM Alexa 488, A-21042, goat anti-rabbit Alexa 488, A11070 [Invitrogen, Molecular Probes]), diluted 1∶1000 in blocking solution. Between incubations samples were washed with PBST (PBS with 0.05% Tween-20 [Sigma]). For DNA staining samples were incubated with 10 µg/ml Hoechst 33342 (Sigma). Samples were mounted with DAKO Cytomation, S3023 (DAKO). Immunostained cells were examined on a confocal Leica TCS SP5 microscope with 20× air and 63× oil immersion objectives. UV-laser (405), Ar-laser (488) and HeNe-laser (633 nm) were used to excite the fluorophores. Images were acquired using the Leica TCS SP5 software.

### Immunoblotting

Trypsin-EDTA-dislodged cells were lysed in RIPA buffer (PBS supplemented with 1% NP-40, 0.5% sodium deoxycholate, 0.1% SDS) supplemented with Protease Inhibitor Cocktail, # 11836170001 (Roche). Protein concentrations were determined with Bradford reagent, B6916 (Sigma), and equal amounts of total protein were resolved by 10% SDS-polyacrylamide gel electrophoresis, transferred to PVDV membranes (Amersham), probed with primary antibodies (same as immunofluorescence, and anti-beta Actin, #4967 [Cell Signaling], anti-Sox-2, ab15830, [Abcam], anti-Nanog, ab21603, [Abcam]), anti-STAT3, 9132, anti-phospho-tyrosine 705 STAT3, 9131, GAPDH, 2118, [Cell Signaling], secondary goat anti-mouse HRP-conjugated, #1858413 (Pierce), goat anti-rabbit HRP-conjugated, #1858415 (Pierce), and detected by chemiluminescence using the ECL kit (Pierce) and Kodak film.

### Real-time PCR Analysis

RNA was extracted by GeneElute Total RNA purification Kit (Sigma), and residual genomic DNA was removed by DNase I treatment (DNA amplification grade, Sigma). RNA was reverse transcribed using random hexamers and Moloney murine leukemia virus reverse transcriptase (Superscript II; Invitrogen). The real-time PCR approach used the SYBR green method in a 96-well plate format using a iQ5 BioRad cycler. Reactions contained 7.5 µl 2× SYBR MasterMix (Qiagen), primer (100 nM), and template in a total volume of 15 µl. The thermal profile used for amplification was 95°C for 8 min followed by 50 cycles of 95°C for 15 sec, 58°C for 20 sec, and 72°C for 40 sec. At the end of the amplification phase, a melting-curve analysis was carried out on the products formed. Gene expression was normalized to *β-actin* mRNA expression. The method of Livak and Schmittgen [31] was applied to compare gene expression levels between groups, using the equation 2^−ΔΔCT^. The primers used are listed in Supplemental Table S1.

## Supporting Information

Figure S1 (A): Immunofluorescence analysis of Oct-4 expression in B10 and mES cells, scale 25 µM; (B): Immunofluorescence analysis of E-cadherin expression in B10L cell line, scale 25 µM; giant trophoblast cells (asterisk) (C): Western blot analysis of Sox-2 expression in mES cells (1), Mouse Embryonic Fibroblasts (2), C5E passage 5 (3), C5E passage 7 (4), B10L (5), Actin was used as internal control.
(0.49 MB TIF)

Figure S2 Rat preimplantation embryos after injections of labelled B10L cells. The positions of cells integrated into the trophectoderm are shown by arrrow. Images were taken at confocal (A-G) and epifluorescence (H,I) microscopes.
(0.60 MB TIF)

Figure S3 Immunofluorescent analysis of Cdx-2 expression in B10 cells, cultivated in the indicated conditions, merge images of Cdx-2 with Hoechst 33342 are shown, scale 50 µM.
(0.27 MB TIF)

Figure S4 (A): Morphology of C5L cells grown on feeders (MEF) or on plastic with and without LIF; (B): Western blot for phospho-tyrosine 705 STAT-3 in C5L cells after stimulation with LIF; C5L were starved for 12 hours without serum and LIF, and stimulated by 1000 U/ml LIF for 15 minutes, levels of total STAT-3 and GAPDH are shown in the same lysates.
(0.65 MB TIF)

Figure S5 Immunofluorescent analysis for Nanog in B10L and C5E cells. mES cells and rat SD blastocysts are shown as positive controls, nuclei were stained by Hoechst 33342, scale 25 µM.
(0.43 MB TIF)

Figure S6 Morphology and expression of trophoblast and extraembryonic endoderm specific markers in cell lines derived from rat preimplantation embryos. (A): Clump formed by blastocyst (WKY + Fisher) after 8 day cultivation on feeder; outgrowing cells with XEN morphology are shown with a white arrowheads. (B): Morphology of rat cell line derived from preimplantation embryo at passage 4; giant trophoblast cells (asterisk), XEN-like cells (arrow) and tight clumps (arrowhead) can be seen. (C): the expression of Cdx2 and Cytokeratin 7 in a cell line derived from a Fisher rats. (D): expression of laminin B2, collagen 4 in a XEN-like cell line derived from a Fisher rat preimplantation embryo.
(1.31 MB TIF)

Figure S7 Morphology of rat TS-like cell line derived from C5E cell line. (A): phase contrast (left) and differential interference contrast (dic, right) photos of rat TS-like cells, photos were taken with 5× objective, arrow indicates the characteristic rat TS clump. (B): Immunofluorescence analysis for Cdx-2 expression (right) and corresponding phase contrast image (left) (C): Immunofluorescence analysis for Cdx-2 expression (left) and corresponding merged image of nuclei staining with Hoechst 33342 (left). Photos were taken with 40× objective.
(1.17 MB TIF)

Table S1 Summary of real time RT-PCR primers.
(0.01 MB TIF)
