# Supplementary figures and images for: Characterization of Trophoblast and Extraembryonic Endoderm Cell Lineages Derived from Rat Preimplantation Embryos
Source: PLoS One. 2010 Mar 29;5(3):e9794. doi: 10.1371/journal.pone.0009794 (PMC2848026; doi:10.1371/journal.pone.0009794)

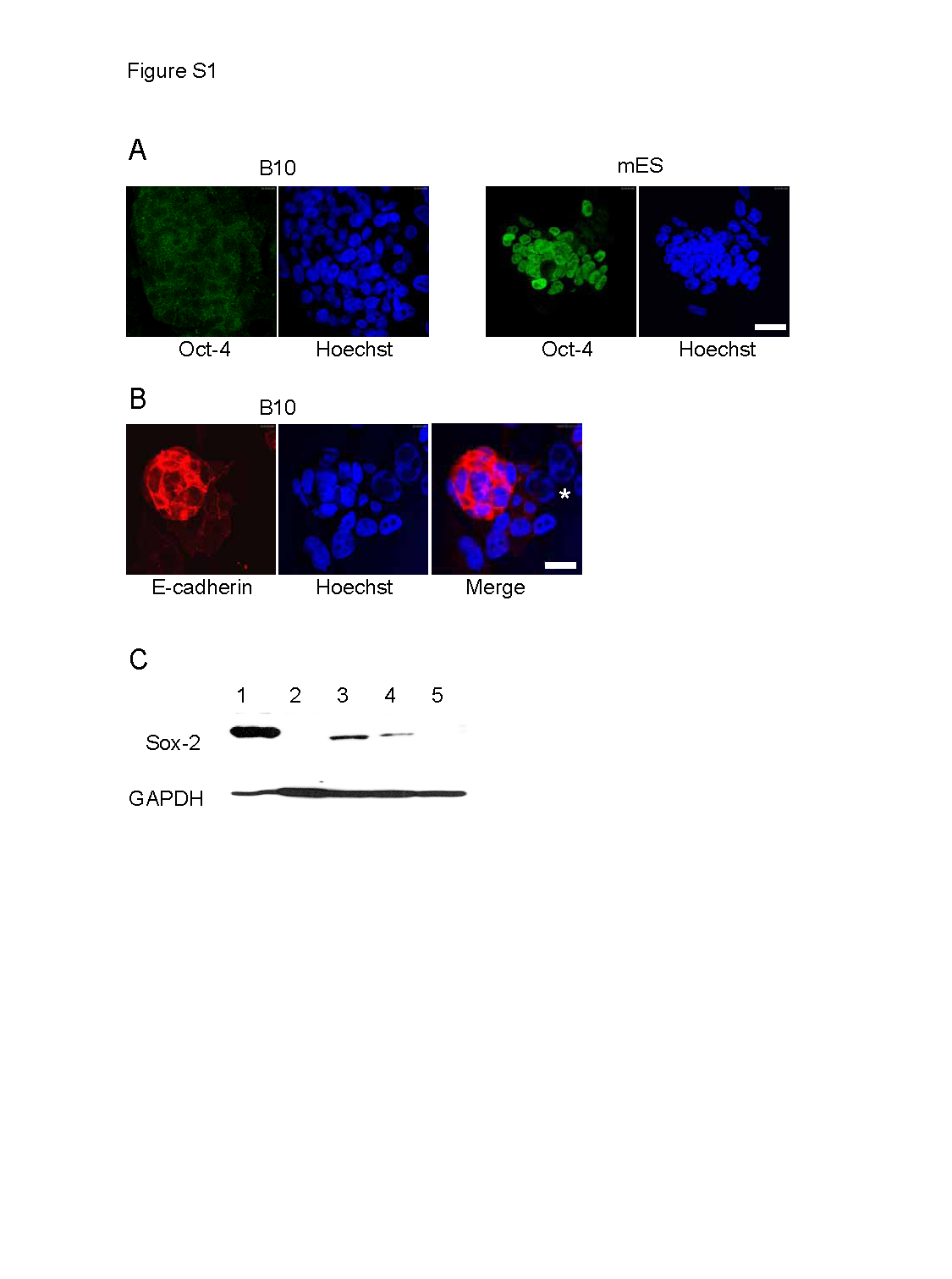

Supplement: Figure S1 — (A): Immunofluorescence analysis of Oct-4 expression in B10 and mES cells, scale 25 µM; (B): Immunofluorescence analysis of E-cadherin expression in B10L cell line, scale 25 µM; giant trophoblast cells (asterisk) (C): Western blot analysis of Sox-2 expression in mES cells (1), Mouse Embryonic Fibroblasts (2), C5E passage 5 (3), C5E passage 7 (4), B10L (5), Actin was used as internal control. (0.49 MB TIF) [file pone.0009794.s001.tif]

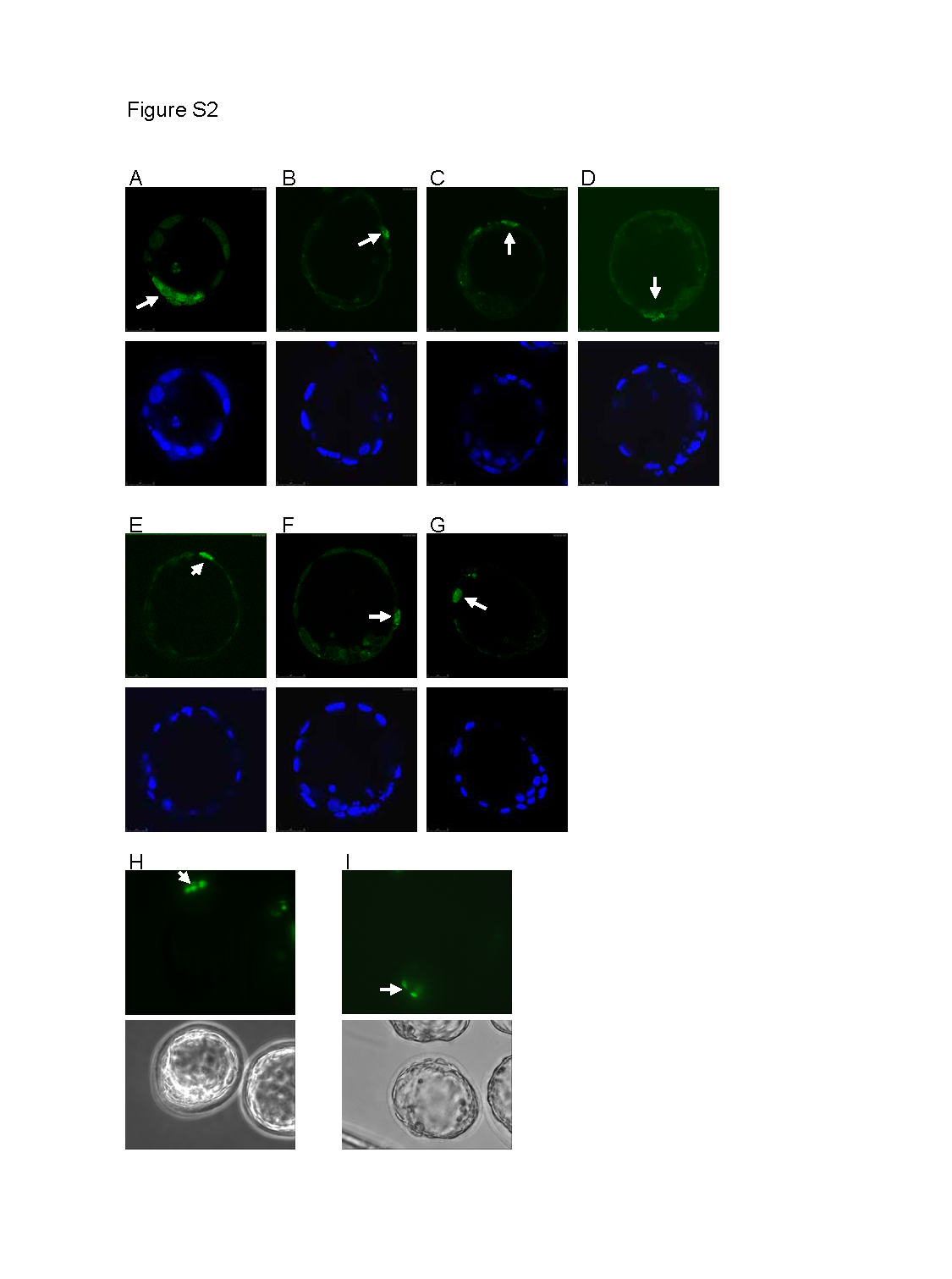

Supplement: Figure S2 — Rat preimplantation embryos after injections of labelled B10L cells. The positions of cells integrated into the trophectoderm are shown by arrrow. Images were taken at confocal (A-G) and epifluorescence (H,I) microscopes. (0.60 MB TIF) [file pone.0009794.s002.tif]

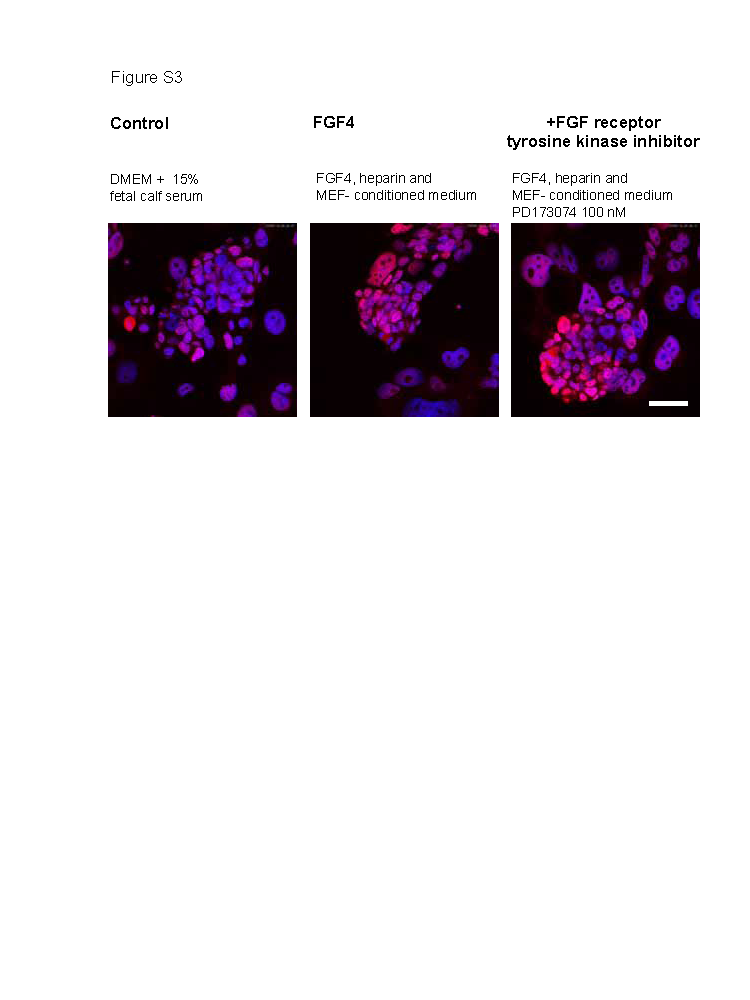

Supplement: Figure S3 — Immunofluorescent analysis of Cdx-2 expression in B10 cells, cultivated in the indicated conditions, merge images of Cdx-2 with Hoechst 33342 are shown, scale 50 µM. (0.27 MB TIF) [file pone.0009794.s003.tif]

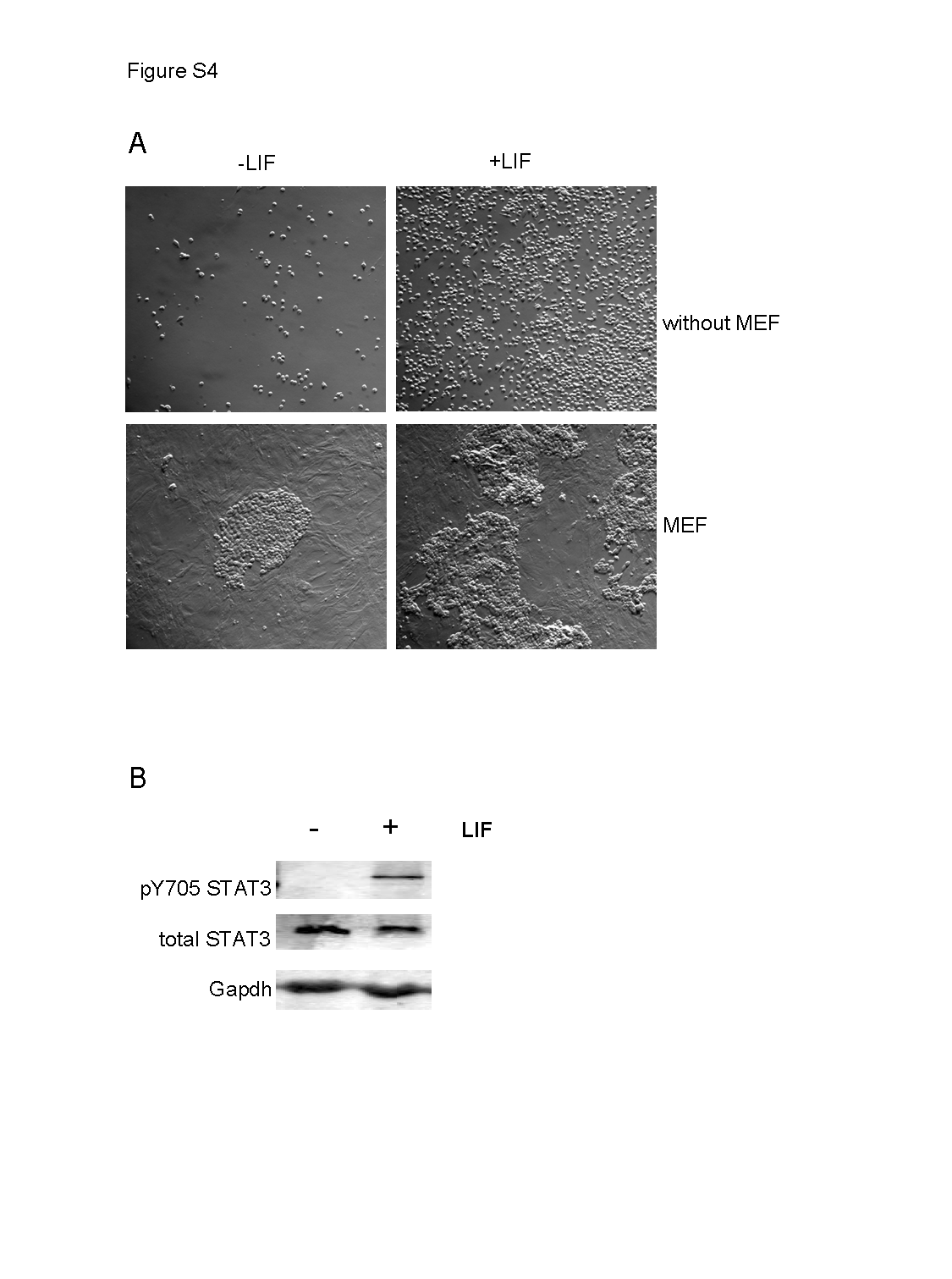

Supplement: Figure S4 — (A): Morphology of C5L cells grown on feeders (MEF) or on plastic with and without LIF; (B): Western blot for phospho-tyrosine 705 STAT-3 in C5L cells after stimulation with LIF; C5L were starved for 12 hours without serum and LIF, and stimulated by 1000 U/ml LIF for 15 minutes, levels of total STAT-3 and GAPDH are shown in the same lysates. (0.65 MB TIF) [file pone.0009794.s004.tif]

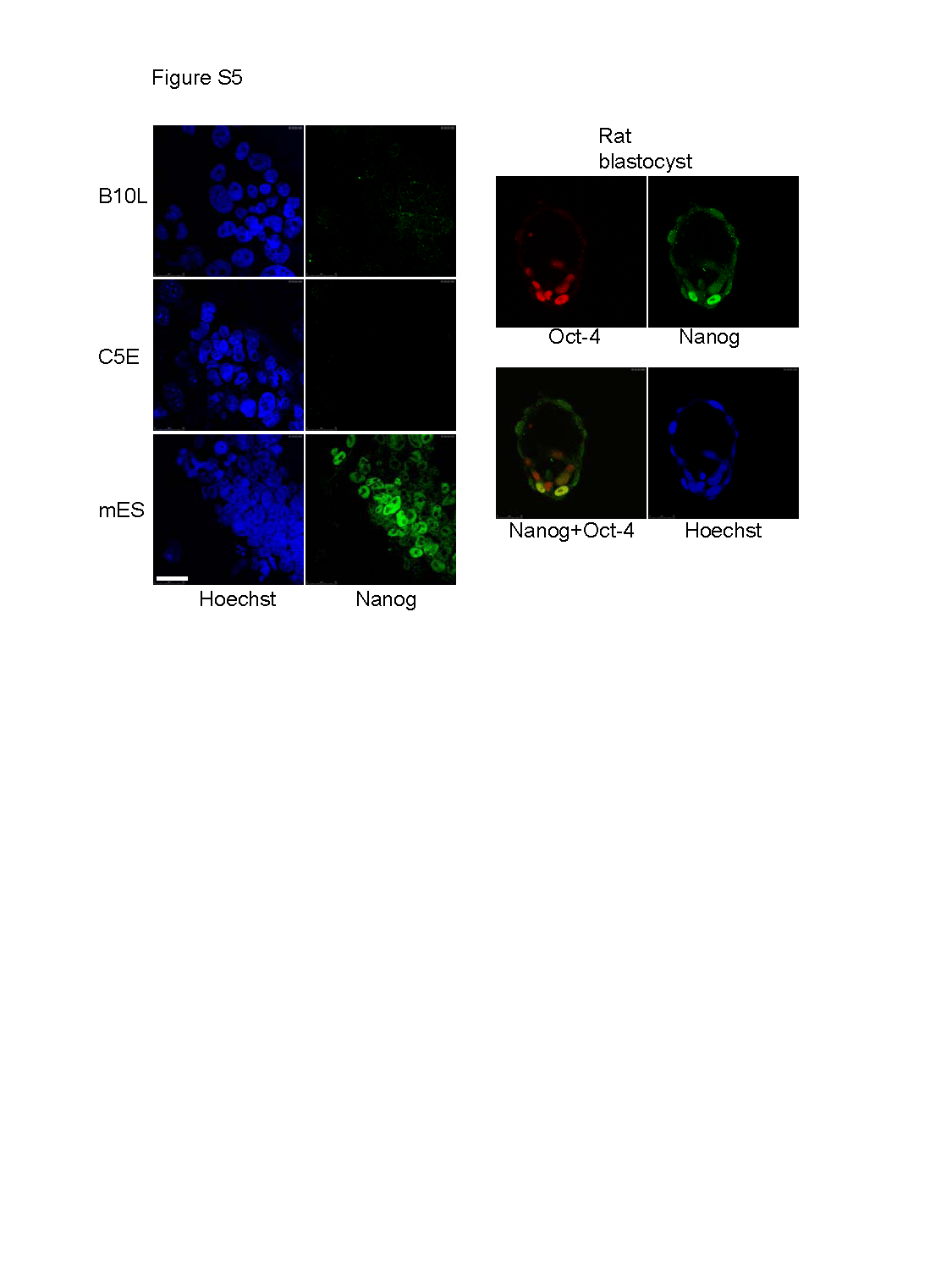

Supplement: Figure S5 — Immunofluorescent analysis for Nanog in B10L and C5E cells. mES cells and rat SD blastocysts are shown as positive controls, nuclei were stained by Hoechst 33342, scale 25 µM. (0.43 MB TIF) [file pone.0009794.s005.tif]

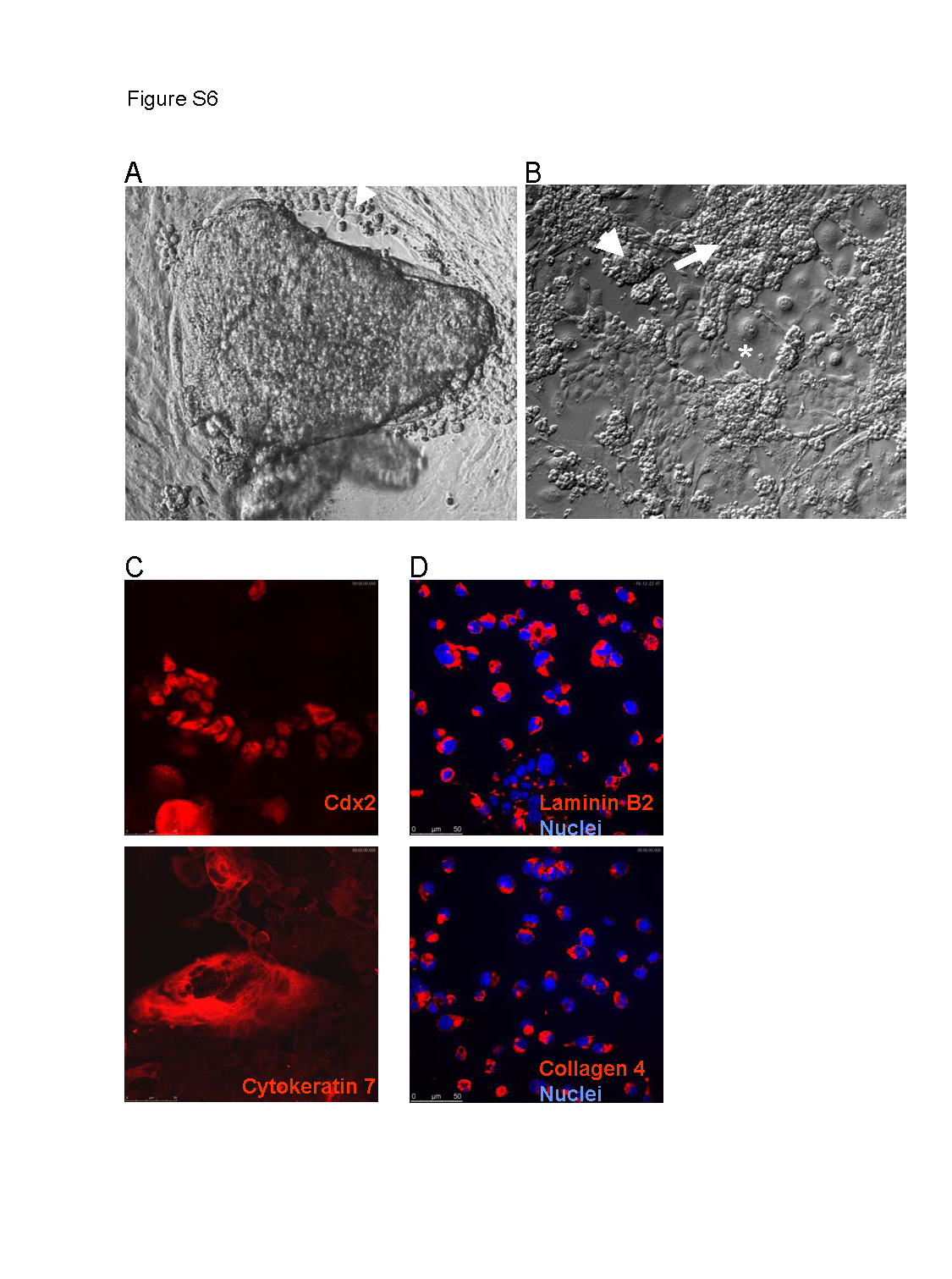

Supplement: Figure S6 — Morphology and expression of trophoblast and extraembryonic endoderm specific markers in cell lines derived from rat preimplantation embryos. (A): Clump formed by blastocyst (WKY + Fisher) after 8 day cultivation on feeder; outgrowing cells with XEN morphology are shown with a white arrowheads. (B): Morphology of rat cell line derived from preimplantation embryo at passage 4; giant trophoblast cells (asterisk), XEN-like cells (arrow) and tight clumps (arrowhead) can be seen. (C): the expression of Cdx2 and Cytokeratin 7 in a cell line derived from a Fisher rats. (D): expression of laminin B2, collagen 4 in a XEN-like cell line derived from a Fisher rat preimplantation embryo. (1.31 MB TIF) [file pone.0009794.s006.tif]

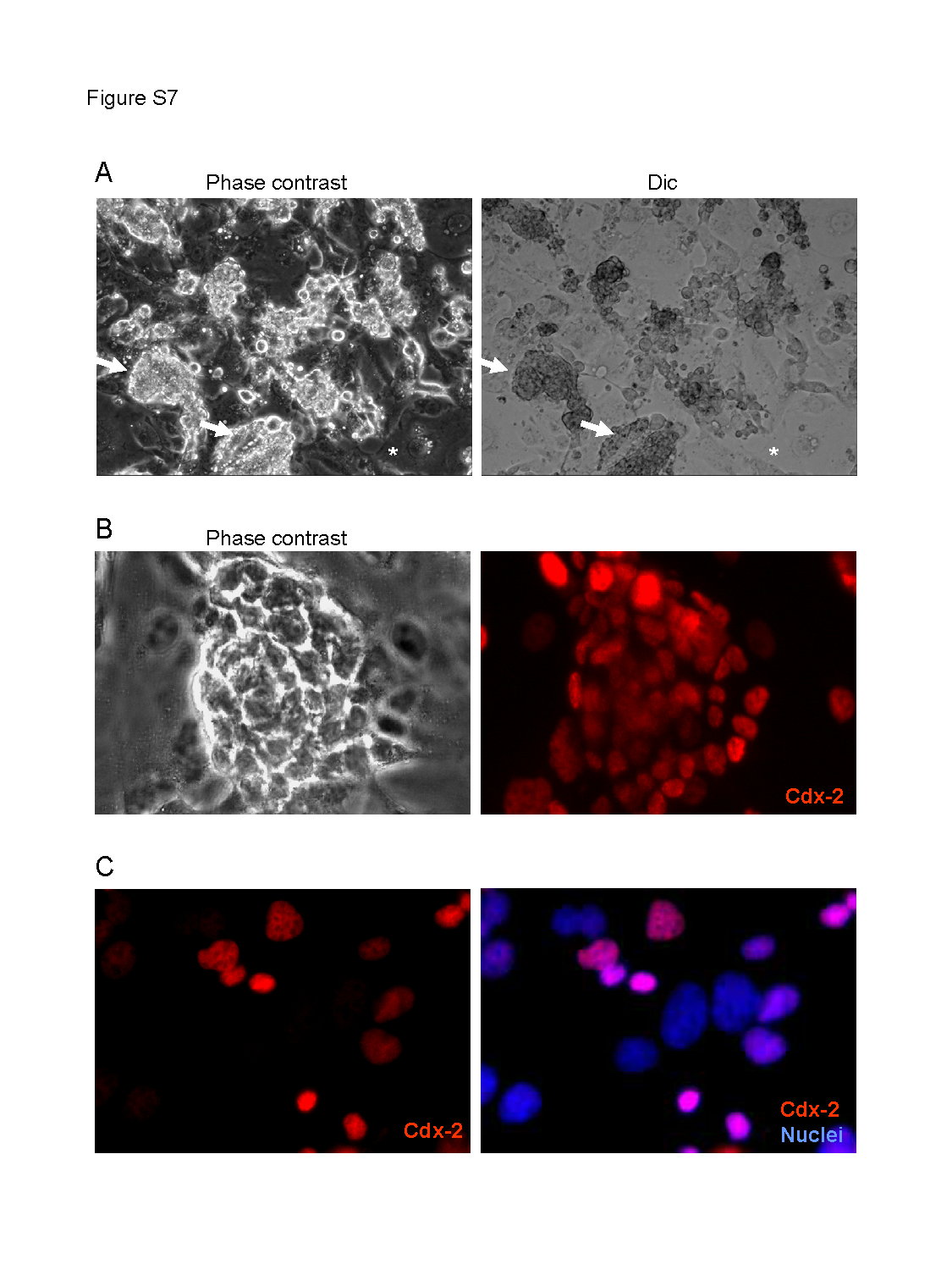

Supplement: Figure S7 — Morphology of rat TS-like cell line derived from C5E cell line. (A): phase contrast (left) and differential interference contrast (dic, right) photos of rat TS-like cells, photos were taken with 5× objective, arrow indicates the characteristic rat TS clump. (B): Immunofluorescence analysis for Cdx-2 expression (right) and corresponding phase contrast image (left) (C): Immunofluorescence analysis for Cdx-2 expression (left) and corresponding merged image of nuclei staining with Hoechst 33342 (left). Photos were taken with 40× objective. (1.17 MB TIF) [file pone.0009794.s007.tif]
